# The DREB A-5 Transcription Factor ScDREB5 From *Syntrichia caninervis* Enhanced Salt Tolerance by Regulating Jasmonic Acid Biosynthesis in Transgenic Arabidopsis

**DOI:** 10.3389/fpls.2022.857396

**Published:** 2022-04-06

**Authors:** Jinyuan Liu, Ruirui Yang, Yuqing Liang, Yan Wang, Xiaoshuang Li

**Affiliations:** ^1^Xinjiang Key Laboratory of Biological Resources and Genetic Engineering, College of Life Science and Technology, Xinjiang University, Urumqi, China; ^2^State Key Laboratory of Desert and Oasis Ecology, Xinjiang Institute of Ecology and Geography, Chinese Academy of Sciences, Urumqi, China; ^3^University of Chinese Academy of Sciences, Beijing, China

**Keywords:** DREB transcription factor, *Syntrichia caninervis*, salt stress, jasmonic acid (JA) biosynthesis, ROS scavenging ability

## Abstract

Salinity is a major limiting factor in crop productivity. Dehydration-responsive element-binding protein (DREB) transcription factors have been widely identified in a variety of plants and play important roles in plant stress responses. Studies on DREBs have primarily focused on the A-1 and A-2 DREB groups, while few have focused on the A-5 group. In this study, we concentrated on *ScDREB5*, an A-5b type *DREB* gene from the desiccation-tolerant moss *Syntrichia caninervis*. *ScDREB5* is a transcription factor localized to the nucleus that exhibits transactivation activity in yeast. Ectopic *ScDREB5* expression in *Arabidopsis thaliana* increased seed germination and improved seedling tolerance under salt stress. *ScDREB5*-overexpression transgenic Arabidopsis lines showed lower methane dicarboxylic aldehyde (MDA) and hydrogen peroxide (H_2_O_2_) contents, but higher peroxidase (POD), superoxide dismutase (SOD), and catalase (CAT) activities compared to wild plants. Moreover, the transcriptional levels of stress marker genes, including *RD29B*, *COR47*, *LEA6*, *LEA7*, *ERD1*, *P5CS1*, and salt overly sensitive (SOS) genes (*SOS1*, *SOS2*, and *SOS3*), were upregulated in the transgenic lines when subjected to salt treatment. Transcriptome and real-time quantitative PCR (RT-qPCR) analyses indicated that transgenic lines were accompanied by an increased expression of jasmonic acid (JA) biosynthesis genes, as well as a higher JA content under salt stress. Our results suggest that *ScDREB5* could improve salt tolerance by enhancing the scavenging abilities of reactive oxygen species (ROS), increasing JA content by upregulating JA synthesis gene expression, regulating ion homeostasis by up-regulating stress-related genes, osmotic adjustment, and protein protection, making *ScDREB5* a promising candidate gene for crop salt stress breeding.

## Introduction

In nature, plants are constantly challenged by adverse abiotic environmental conditions, such as drought, heat, cold, nutrient deficiencies, and excess salt or toxic metal levels in the soil. These abiotic stresses limit the global utilization of arable lands and negatively affect crop productivity (Zhang et al., 2021). Salinity is one of the most deteriorating of these stresses because it affects approximately 20% of irrigated agricultural land and one-third of the agricultural productivity globally (Shah et al., 2021). High salinity causes ionic, osmotic, and oxidative stresses to plants (Song et al., 2021). To protect against the detrimental effects of environmental stresses, plants have gradually evolved appropriate response mechanisms, in which gene expression regulation plays an important role (Gururani et al., 2015). Transcription factors are critical abiotic stress tolerance regulators (Sakuma et al., 2006a); for example, APETALA2/ethylene responsive factor (AP2/ERF), one of the largest plant transcription factor families containing a highly conserved AP2 domain, plays a key role in plant development and stress responses (Xu et al., 2011; Chandler, 2018). Based on the number of AP2 and B3 domains, the AP2/ERF family is comprehensively divided into the AP2, ERF, related to ABI3 and VP1 (RAV), dehydration-responsive element-binding protein (DREB), and Soloist subfamilies (Sakuma et al., 2002; Nakano et al., 2006; Giri et al., 2014). Among these, the DREB subfamily is mainly involved in drought, high salinity, cold, and heat stress responses (Sakuma et al., 2006b; Qin et al., 2007; Matsukura et al., 2010; Mizoi et al., 2013) and has been extensively studied in many plants (Mizoi et al., 2012). The DREB subfamily is further divided into six subgroups, A-1–A-6. The functions of A-1 and A-2 have been thoroughly studied (Agarwal et al., 2017), while the functions and mechanisms of A-5 type genes have received limited attention and remain unclear, although A-5 type DREB has great potential.

In recent years, most reports have shown that A-5 DREB transcription factors can improve stress resistance in plants. In potato plants, low temperature, drought, and abscisic acid (ABA) induced *StDREB2*, an overexpression of which could enhance the salt stress tolerance in transgenic *Arabidopsis thaliana* (Bouaziz et al., 2012). Subsequent studies have shown that an overexpression of *StDREB2* enhanced drought stress tolerance in cotton (El-Esawi and Alayafi, 2019); in soybean plants, *GmDREB2* was induced by drought, salt, low temperature, and ABA treatments, which showed drought and salt stress tolerances in transgenic Arabidopsis (Chen et al., 2007); overexpression of *GmDREB3* enhanced the resistance of transgenic Arabidopsis to cold, drought, and salt stresses (Chen et al., 2009); overexpression of *PpDBF1* has increased salt, drought, and cold stress tolerances in transgenic tobacco plants (Liu et al., 2007); and overexpression of *HhDREB2* in Arabidopsis resulted in enhanced tolerance to salt and drought stresses (Ma et al., 2014). All these studies indicated that A-5 *DREB* genes are good resistance gene candidates for crop resistance breeding; thus, it is meaningful to study the biological functions of A-5 *DREB* genes from different plants.

Mosses are one of the oldest species in the transition from aquatic to terrestrial plants (Oliver et al., 2000), and because of their high oxygen content, intense atmospheric radiation, and drastic temperature changes on land, mosses have evolved special physiological and biochemical characteristics to tolerate multiple stresses (Wu et al., 2012; Pan et al., 2016). *S. caninervis*, a desiccation-tolerant moss, is a dominant species in biological soil crusts in the Gurbantunggut Desert of Northwestern China (Wang et al., 2020a), and was proven to be a category 1 (A) desiccation tolerance (DT; Wood, 2007). To adapt to harsh desert environments, *S. caninervis* has evolved multiple stress tolerances. Many stress-related genes have been identified from *S. caninervis*, making it a well-studied model for plant DT mechanism studies and stress-related gene identification (Yang et al., 2012, 2015; Li et al., 2015, 2016, 2017).

In a previous study, we found that A-5 *DREB* genes were largely expanded in *S*. *caninervis*, indicating their importance in stress responses (Li et al., 2017). Ten A-5 *DREB* genes (*ScDREB1*–*ScDREB10*) were screened and cloned based on the *S. caninervis* dehydration-rehydration transcriptome (Li et al., 2016). Among these, an overexpression of *ScDREB8* could improve salt tolerance in Arabidopsis (Liang et al., 2017), while an overexpression of *ScDREB10* significantly increased the drought and salt tolerance of transgenic Arabidopsis at both the germination and seedling stages (Li X. et al., 2019). In the present study, we further characterized the function of *ScDREB5*. Our results showed that *ScDREB5* is located in the nucleus and possesses transactivation activity. We overexpressed *ScDREB5* in transgenic Arabidopsis to evaluate its role in salt stress responses. Our findings suggest that *ScDREB5* is a positive regulator of the salt-stress response that improves salt tolerance by enhancing reactive oxygen species (ROS) scavenging abilities, upregulating stress-responsive genes, and modulating jasmonic acid (JA) biosynthesis in plants under salt stress.

## Materials and Methods

### Plant Samples and Treatments

Ecotype colombia of Arabidopsis (Col-0) was used as the wild type (WT), and the genetic background for transgenic plants. Plants were grown in a growth chamber at 22°C using 16/8 h light/dark photocycles (approximately 100 μmol m^–2^s^–1^ constant light, 60% relative humidity).

Seven-day-old seedlings, grown under normal conditions, were carefully transferred to a Murashige and Skoog (MS) medium (control) or a MS medium containing 150 mM NaCl and incubated for 7 d. Two transgenic lines with three replicates were used for salt stress experiments, with at least 20 seedlings were used for each replicate. Whole plants were harvested for physiological index analysis, JA content measurement, real-time quantitative PCR (RT-qPCR), and RNA sequencing.

### DNA/Protein Sequence and Phylogenetic Analyses

The protein sequences of classic DREBs from the model plants *A*. *thaliana*, *Oryza sativa*, *Vaccinium vitis-idaea*, *Malus sieversii*, *Zea mays*, *Glycine max*, and *Gossypium hirsutum* were retrieved from the National Center for Biotechnology Information (NCBI) Entrez database^1^. Multiple sequence alignments of amino acid sequences from different species were performed using ClustalW, and phylogenetic trees were constructed with the neighbor-joining (NJ) method and a bootstrap test with 1000 replicates using MEGA7 software for Poisson correction and pairwise deletion (Kumar et al., 2016). Motif prediction of *ScDREB5* and other classic DREB proteins was performed using MEME (Bailey et al., 2015).

### Subcellular Localization Analysis

The open reading frame (ORF) for *ScDREB5* without a stop codon was amplified from pMD18-T-*ScDREB5* positive plasmids using gene-specific primers containing an *Sma*I restriction site, and the PCR product was fused to the N-terminus of the green fluorescent protein (GFP) gene driven by the CaMV 35S promoter in the pBI121 vector using an in-fusion PCR cloning system (Clontech, Mountain View, CA, United States). The primers used in this experiment are listed in Supplementary Table 1. Both the transient expression of the *35S:ScDREB5-GFP* fusion and *35S:GFP* control plasmids were introduced into living onion epidermal cells *via Agrobacterium*-mediated infiltration for transient transformation (Sun et al., 2007). The transformed cells were cultured in 1/2 Murashige and Skoog (MS) medium at 22°C ± 2°C in the dark for 24 h and visualized using confocal laser scanning microscopy LSM800 (Zeiss, Jena, Germany).

### Analysis of Transactivation Activity of ScDREB5

The coding sequence of *ScDERB5* was fused in frame with the GAL4 DNA-binding domain (pGBKT7 vector) to produce the BD-ScDREB5 fusion construct using the in-fusion PCR cloning system (Clontech), and the full-length ORF of *ScDREB5* was amplified from pMD18-T plasmids by PCR using gene-specific primers containing *Eco*RI and *Bam*HI restriction sites, and the PCR product was inserted into the *Eco*RI/*Bam*HI pGBKT7 vector. The construct was subsequently introduced into yeast Y2H gold cells (Clontech). The yeast positive transformants were adjusted to an OD600 of 2.0, and the yeast cells were then serially diluted 10-fold and dropped with 2 μL of synthetic dropout (SD) medium without tryptophan (SD/-Trp), without tryptophan and histidine (SD/-Trp/-His), and with SD/-Trp/-His plates containing x-α-gal (SD/-Trp/-His + x-α-gal). Yeast cells expressing the pGBKT7 empty vector or expressing GAL4 were used as negative and positive controls, respectively. The plates were incubated at 30°C for 2–4 d before photographing. Primer information is listed in Supplementary Table 1.

### Generation of ScDREB5-Overexpressing Arabidopsis

The *35S:ScDREB5-GFP* was introduced into WT Arabidopsis plants using the floral dip method (Clough and Bent, 1998). T_1_ to T_3_ generation seeds of transgenic plants were selected on MS medium containing 80 mg L^–1^ hygromycin. The hygromycin-resistant T_1_ seedlings were tested by PCR analysis and sequencing, and the T_3_ homozygous lines produced from the T_1_ plants expressing the *ScDREB5* gene. We obtained 18 transgenic lines in total (the positive tests of all the T_3_ seedings lines by PCR analysis were provided and showed in Supplementary Figure 1A). The ScDREB5 had the higher expression in line 2 and line 3 in different T_3_ transgenic lines (Supplementary Figure 1B), which were selected for further functional analysis.

### Evaluation of the Salt Stress Tolerance of Transgenic Arabidopsis at Germination Stage

To determine the sensitivity of the seed germination process to salt stress, Arabidopsis seeds of WT and transgenic plants were surface-sterilized and placed on MS agar plates (control) or MS agar plates supplied with 150 mM NaCl (salt stress). Fifty seeds per line with three replicates were used for the stress germination experiments. The seeds were incubated at 4°C for 2 d and then transferred to a growth chamber at 22 ± 2°C under a 16/8 h light/dark cycle. For germination rate statistics, the number of seeds that germinated were recorded every 24 h for 7 d. Seeds were considered to have germinated when the radicles were 1 mm long. Germination percentages were recorded as the number of seeds that germinated out of the total number of seeds tested.

### Phenotype and Physiological Index Analysis of Transgenic Arabidopsis

After 7 d of salt stress treatment, the phenotype of plants was photographed under normal and stress conditions, and the root length, fresh weight, and number of lateral roots were measured. Then, these seedlings were collected and used for diaminobenzidine (DAB) and nitrotetrazolium blue chloride (NBT) staining, and seedlings were harvested by immediate flash freezing in liquid nitrogen to measure physiological indices. WT and transgenic plants (100 mg fresh weight) were ground with ice-cold 0.1 mol L^–1^ potassium phosphate buffer (pH 7.4; 1:9 w/v) and clarified by centrifugation at 8000 × *g* for 10 min at 4°C. The supernatants were harvested for further physiological analysis, and hydrogen peroxide (H_2_O_2_) levels, methane dicarboxylic aldehyde (MDA) content, superoxide dismutase (SOD, EC 1.15.1.1), catalase (CAT, EC 1.11.1.6), and peroxidase (POD, EC 1.11.1.7) activities were measured using assay kits (kit Numbers. A064-1, A003-3, A001-3, A084-3, A007-1, Nanjing Jian Cheng Bioengineering Institute, China), respectively.

Jasmonic acid was extracted and quantified by Suzhou Comin Biotechnology Co., Ltd., by high-performance liquid chromatography apparatus Rigol L3000 (Rigol, Beijing, China).

### Transcriptomic Analysis

The L2 was chosen for the transcriptomic profiling analysis due to better stress tolerant phenotypes (great fresh weight, more number of lateral roots). L2 and WT plant samples were collected before and after salt stress with three biological replicates, total twelve samples were used for RNA isolation. RNA purity and concentration were then examined using a NanoPhotometer® spectrophotometer; RNA integrity and quantity were measured using the RNA Nano 6000 Assay Kit of the Bioanalyzer 2100 system. Total RNA was used as the input material for library preparation. RNA sequencing analysis was performed by Novogene Corporation (Beijing, China). After the unique molecular identifier (UMI) library preparation and pooling of different samples, the samples were subjected for Illumina sequencing. The pearson’s correlation between samples showed in Supplementary Figure 2. Then, the fragments per kilobase of exon model per million mapped reads (FPKM) of each gene was calculated based on the length of the gene and read counts mapped to this gene. Differential expression analysis was performed using DESeq R package (1.10.1). Genes with | log_2_(fold change) | > 1 and an adjusted *P*-value < 0.05, were considered differentially expressed.

### RT-qPCR Assay

RNA was extracted using a Plant RNA Kit (OMEGA, Guangzhou, China). First-strand cDNA was synthesized using 1 μg of RNA with the PrimeScript RT reagent Kit (TaKaRa, Tokyo, Japan). RT-qPCR was performed on a CFX-96 Real-Time System (Bio-Rad, Hercules, CA, United States) with SYBR Premix Ex Taq II (TaKaRa). The *AtTubulin* (*AT1G50010*) gene of Arabidopsis was used as a reference for RT-qPCR normalization. The PCR conditions were as follows: initial denaturation step of 30 s at 95°C, 40 cycles of PCR (95°C for 5 s, 58–60°C for 30 s). Three biological and technological replicates were performed for each sample. The relative expression of the detected genes was calculated using the 2^–ΔΔ*Ct*^ method. The expression levels of classic stress-responsive genes (*AtRD29B*, *AtCOR47*, *AtLEA6*, *AtLEA7*, *AtERD1*, and *AtP5CS1*), salt stress-related salt overly sensitive (SOS) genes (*AtSOS1*, *AtSOS2*, and *AtSOS3*), and JA biosynthesis genes (*AtDAD1*, *AtDGL*, *AtDALL3*, *AtLOX3*, *AtLOX4*, *AtAOS*, *AtAOC3*, *AtOPR3*, *AtJMT*, *AtYCYP94C1*, and *AtCYP9483*) were investigated using RT-qPCR. The primers used for the RT-qPCR analysis are listed in Supplementary Table 2.

### Statistical Analysis

One-way analysis of variance (ANOVA) was used for statistical analyses with the SPSS software (version 19.0; IBM Corp., Armonk, NY, United States). All data in this study represent the mean values ± standard error (SE) of at least three replicates. We used the least significant difference (LSD) multiple comparison tests to identify the significance level, and *p < 0.05 was considered significant, while ^**^p < 0.01 was considered highly significant. All figures were generated using GraphPad Prism 9 and Adobe Illustrator CS software. Heatmaps were generated using TBtools (Chen et al., 2020).

## Results

### Characterization and Phylogenetic Analysis of ScDREB5

**FIGURE 1:**
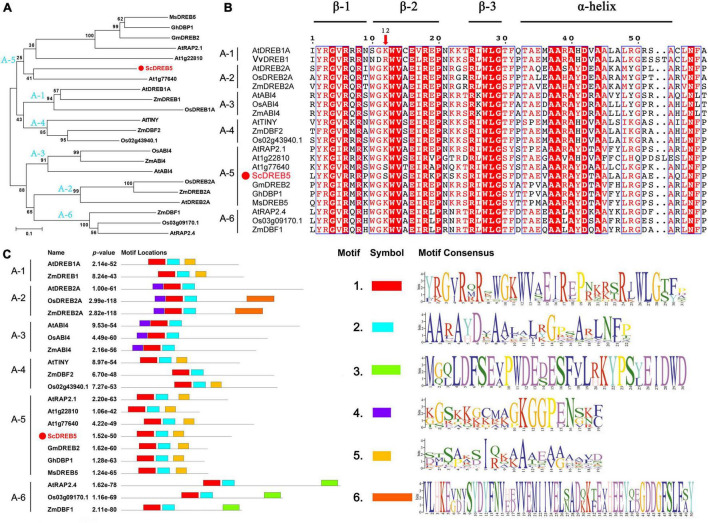
Phylogenetic analysis, sequence alignment and motif prediction of ScDREB5. **(A)** Phylogenetic analysis of ScDREB5 with DREBs from different plant species. A phylogenetic tree was constructed using the neighbor-joining method with Poisson correction (pairwise deletion), and bootstrap values from 1000 replicates were used to assess the robustness of the tree. **(B)** Multiple sequence alignment of ScDREB5 with other classic DREB proteins from different plants. **(C)** Motif prediction of ScDREB5 with other classic DREB proteins. The red color highlights the same amino acid sequence. All amino acid sequences were retrieved from GenBank: *S. caninervis* ScDREB5 (AMT92113.1); *A*. *thaliana* AtDREB1A (BAA33791), AtDREB2A (BAA33794), AtABI4 (ABE65896.1), AtTINY (NP_197953.1), AtRAP2.1 (OAP00017.1), AT1G22810 (ABD57508.1) AT1G77640 (AAT06448.1), AtRAP2.4 (NP_177931.1); *O*. *sativa*, OsDREB2A (AFB77198), OsABI4 (C7J2Z1.1), Os02g43940.1 (XP_015626317.1), Os03g09170.1 (XM_015773644.2); *V*. *vitis-idaea* VvDREB1 (AFC89543.1); *Z*. *mays* ZmDREB2A (PWZ08831.1), ZmABI4 (KJ727241.1), ZmDBF2 (AAM80485.1), ZmDBF1 (AAM80486.1); *M*. *sieversii* MsDREB5 (AFM84627.1), *G. max* GmDREB2 (ABB36645.1); *G. hirsutum* GhDBP1 (AAO43165.1).

The *ScDREB5* gene has been cloned previously (Li et al., 2016), full length of *ScDREB5* is 889 bp, containing an ORF of 612 bp and encoded a predicted polypeptide of 203 amino acids with a putative molecular mass of 22.29 kDa with an isoelectric point of 5.97. ScDREB5 shared the highest sequence similarity with AT1G77640 in Arabidopsis (55% identity) with full-length predicted protein. Phylogenetic analysis showed that ScDREB5 clustered with the A-5 DREB proteins (Figure 1A). Sequence alignment analysis showed that ScDREB5 shared a conserved AP2 domain with three β-sheets and an α-helix, while having little sequence similarity outside the AP2 domain compared to other DREBs (Figure 1B). Altogether, 18 out of the 59 amino acids in AP2 domains, including the residues G-4, R-6, R-8, W-13, V-14, E-16, R-18, P-20, R-25, W-27, L-28, G-29, A-37, A-38, A-40, D-42, A-44 and N-59, were completely conserved among the 21 representative A-1–A-6 type DREB proteins from various plants. The 12th amino acid is an S in ScDREB5 and AT1G77640 proteins; however, it is a K for other DREB proteins. Motif analysis results showed that, similar to other classic DREB A-5 proteins, ScDREB5 has motif 1 (three β-sheets) and motif 2 (α-helix), which is composed of the AP2 domain, motif 3 seems unique for A-6 group DREBs, motif 4 exists in A-2 and A-3 DREBs, and motif 5 is predominately located in A-5 type DREBs and in some of the A-1 and A-4 DREBs (Figure 1C).

### Subcellular Localization and Transactivation Activity Analysis of the ScDREB5 Protein

**FIGURE 2:**
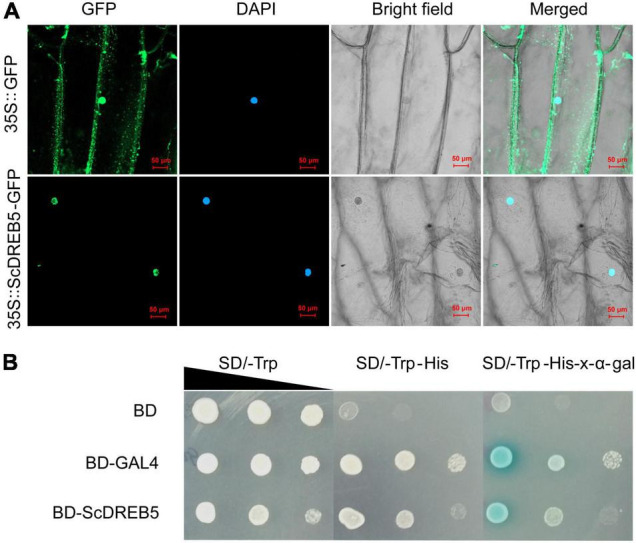
Subcellular localization and transactivation activity analyses of ScDREB5 protein. **(A)** 35S:ScDREB5-GFP and 35S:GFP fusion protein were transiently expressed in onion epidermal cells. After DAPI staining, the transformed cells were examined using confocal microscopy to simultaneously capture GFP (488 nm) and DAPI (405 nm) signals. Left to right: green, GFP fluorescence; blue, nucleus stained with DAPI; bright field; merged, combined fluorescence from GFP and DAPI (scale bar: 50 μm). **(B)** The transcriptional activation ability of ScDREB5 was examined using the yeast one-hybrid assay. Yeast cells Y2H expressing the fusion proteins were cultured and adjusted to an OD600 of 2.0, then series diluted and dropped with 2 μL on nutritional selective mediums SD/-Trp, SD/-Trp/-His and SD/-Trp/-His + x-α-gal. Yeast cells expressing pGBKT7-BD empty vector served as the negative control and those expressing pGBKT7-GAL4 served as the positive control. Photos were taken after incubating at 30°C for 2–4 d.

To understand the characteristics of ScDREB5 as a transcription regulator, we determined its localization and transactivation activity. The subcellular location of the ScDREB5 protein was analyzed by transient expression of the GFP fusion protein introduced into onion epidermal cells. The results revealed that ScDREB5-GFP fusion was co-localized to the nucleus with DAPI stain. In contrast, the control GFP protein was distributed in the nucleus and cytoplasm, indicating that ScDREB5 is a nuclear-localized protein (Figure 2A).

To examine the transactivation activity of ScDREB5, the Y2H system was transformed with the fusion construct pGBKT7-BD-ScDREB5, the negative control pGBKT7-BD, and the positive control pGBKT7-BD-GAL4. The yeast cells containing pGBKT7-BD-ScDREB5 or pGBKT7-BD-GAL4 grew well in the SD/-Trp/-His medium, whereas yeast cells containing the negative control pGBKT7-BD did not grow (Figure 2B). Furthermore, in the presence of x-α-gal, the yeast cells harboring pGBKT7-BD-ScDREB5 and pGBKT7-BD-GAL4 that grew well in the SD/-Trp/-His medium turned blue (Figure 2B). These results indicate that ScDREB5 has transactivation activity in yeast.

### Overexpression of ScDREB5 Improved the Salt Stress Tolerance of Transgenic Arabidopsis

To further evaluate the function of ScDREB5 in plants, we introduced the *ScDREB5* gene into Arabidopsis. We first analyzed the germination rate and phenotype of the transgenic lines (L2 and L3) and WT plants under drought and salt stress. Under drought stress, there was no significant difference in the germination rate and tolerance phenotype between transgenic and WT plants after drought stress (Supplementary Figure 3); therefore, we focused on the salt stress tolerance function in further investigations.

**FIGURE 3:**
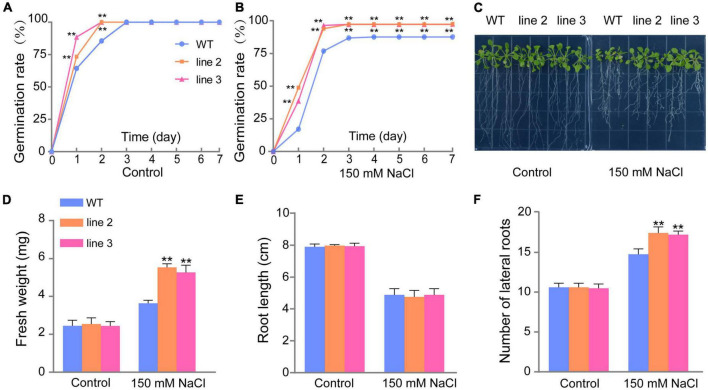
Germination rate, fresh weight, root length, and number of lateral roots comparisons of WT plants and two *ScDREB5* transgenic Arabidopsis lines. **(A,B)** Germination percentages of WT and *ScDREB5*-transformed seeds under normal and salt stress conditions. The germination percentages were calculated as the number of germinated seeds divided by the total number of seeds. Values are means ± SE of three replicates (n = 40–60 seeds). Asterisks indicate statistically significant differences from WT (**P < 0.01). **(C)** Phenotype observation of WT and transgenic plants under normal and salt stress conditions. **(D–F)** Analysis of the fresh weight (mg), root length (cm), and number of lateral roots. Data are displayed with mean ± SE using at least 20 plants per replicates. Significant difference compared with WT plants with the same treatment. LSD multiple comparison test was used: *p < 0.05, **p < 0.01.

Under normal conditions, the seed germination rate and percentage of transgenic lines were faster and higher than those of WT plants during the first 2 days (Figure 3A). Under salt stress, the germination rate of both transgenic lines (L2 and L3) reached 100% on day 3; however, the germination rate of WT plants was only 87.74% until day 7 (Figure 3B). The results indicated that the overexpression of *ScDREB5* increased salt tolerance in Arabidopsis at the germination stage. Additionally, we analyzed the growth of the transgenic lines and WT plants at the seedling stage. Under normal growth conditions, the WT plants and the two transgenic lines had similar phenotypes with comparable fresh weight, root length, and lateral root numbers (Figures 3D–F); however, under salt stress conditions, the transgenic plants had significant higher fresh weight, and the fresh weight of the WT plants was 3.63 mg, while for L2 and L3, they were 5.54 and 5.28 mg, respectively (^**^p < 0.01) (Figure 3D). Although the root length of L2 and L3 were not different from that of the WT plants under salt stress, the lateral root numbers of transgenic plants were significantly higher than that of WT plants (Figures 3E,F) (^**^p < 0.01). These results indicate that overexpression of *ScDREB5* enhances tolerance to salt stress in transgenic plants.

### ScDREB5 Improved Reactive Oxygen Species Scavenging Capability of Transgenic Arabidopsis

**FIGURE 4:**
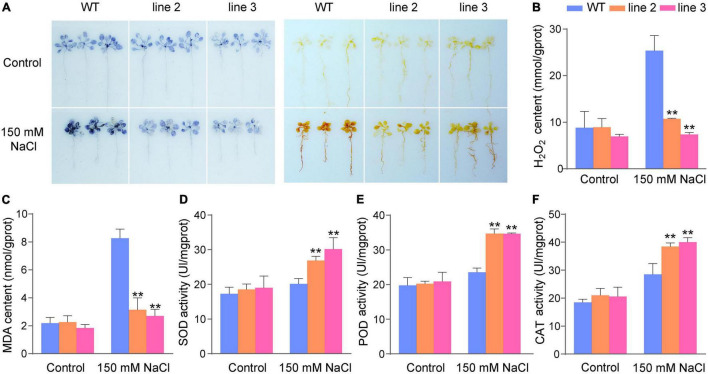
Analysis of ROS level and ROS scavenging ability of transgenic Arabidopsis under salt stress treatment. **(A)** Analysis of ROS production in intact plants by NBT and DAB staining. **(B)** Analysis of H_2_O_2_ level. **(C–F)** Analysis of MDA content, SOD, POD and CAT activities, respectively. All experiments were repeated three times. Data are means ± SE from three independent experiments. Significant difference compared with WT plants with the same treatment, LSD multiple comparison test was used: *p < 0.05, **p < 0.01.

The physiological changes in the transgenic lines and WT were analyzed after salt stress. Abiotic stress can lead to oxidative damage due to increased ROS production. Therefore, it is important for plants to activate antioxidative systems to cope with this oxidative damage. NBT and DAB staining were used to determine the two main ROS species (H_2_O_2_ and superoxide anion, O_2_^–^). As shown in Figure 4A, the staining results showed that there were lighter NBT and DAB staining in transgenic plants than in the WT plants under salt stress conditions; however, there were no significant differences between transgenic and WT plants under normal conditions. This means that transgenic plants have less H_2_O_2_ and O_2_^–^ accumulation than WT plants under salt stress conditions. Accordingly, the H_2_O_2_ levels in *ScDREB5* transgenic plants were significantly lower than those in the WT plants (Figure 4B). We compared the MDA contents between WT and transgenic plants and found that transgenic plants had significantly lower MDA contents after salt stress than WT plants (Figure 4C). Furthermore, transgenic and WT plants showed similar SOD, POD, and CAT activities under normal growth conditions; however, these activities were significantly higher in L2 and L3 than in WT plants under salt stress (Figures 4D–F). In conclusion, our results suggest that *ScDREB5* may enhance the salt resistance of transgenic Arabidopsis by reducing the accumulation of ROS and inducing higher antioxidant activities.

### Transcriptomic Profiling Analysis of Overexpression of ScDREB5 Plants After Salt Stress Treatment

**FIGURE 5:**
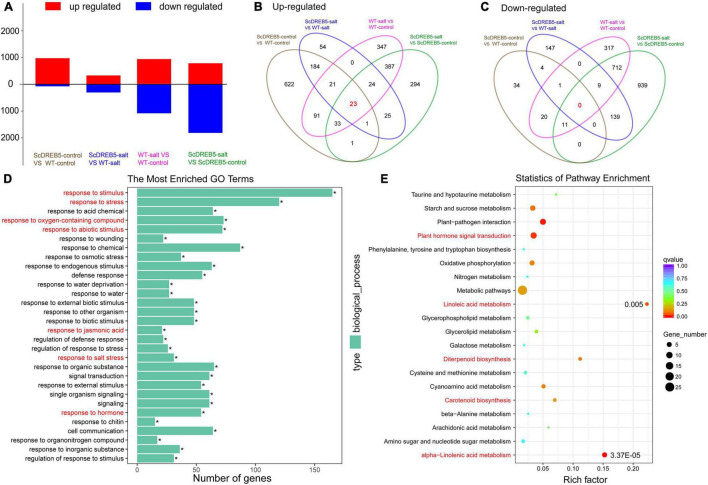
Differentially accumulated transcripts, GO enrichment, and KEGG pathway analysis between *ScDREB5* transgenic and WT plants based on RNA-seq analysis. **(A)** Number of DEGs in WT and the transgenic *ScDREB5* plants under normal and salt stress condition. **(B,C)** Overlapping analyses of up- and downregulated genes induced by *ScDREB5* or salt stress. **(D,E)** GO term and KEGG analyses of the upregulated genes under salt stress (*ScDREB5*-salt vs. WT-salt).

To investigate the molecular regulation mechanism of salt tolerance in *ScDREB5* transgenic plants, we performed RNA-seq of both WT and *ScDREB5* transgenic plants under normal and salt stress conditions. For WT plants, 927 and 1071 differentially expressed genes (DEGs) were up- and downregulated, respectively, after salt stress treatment (WT-salt vs. WT-control); In *ScDREB5* transgenic lines, 789 and 1811 DEGs were up- and downregulated, respectively (*ScDREB5*-salt vs. *ScDREB5*-control) (Figure 5A). Compared to WT plants, more DEGs (977 genes) were up-regulated and fewer DEGs (71 genes) were downregulated in *ScDREB5* transgenic lines under normal conditions (*ScDREB5*-control vs. WT-control), and 333 and 301 DEGs were up- and downregulated in *ScDREB5* transgenic lines under salt stress conditions (*ScDREB5*-salt vs. WT-salt) (Figure 5A). Overlapping analyses of the four comparisons are displayed in Figures 5B,C. Among them, 0 genes were downregulated together (Figure 5C), 23 genes were upregulated together (Figure 5B), and the results of Kyoto Encyclopedia of Genes and Genomes (KEGG) analysis showed that 23 genes were predominately enriched in “cyanoamino acid metabolism” and “plant hormone signal transduction”. To investigate the roles of *ScDREB5* in salt stress tolerance, functional annotation of the upregulated DEGs under salt stress (*ScDREB5*-salt vs. WT-salt) was analyzed by gene ontology (GO) enrichment. For biological process GO terms, “regulation to salt stress”, “response to stimulus”, “response to stress”, “response to abiotic stimulus”, “response to oxygen-containing compound”, “response to JA”, and “response to hormone” were enriched (Figure 5D). Moreover, KEGG pathway analyses revealed that “plant hormone signal transduction”, “alpha-linolenic acid metabolism”, “linoleic acid metabolism”, “diterpenoid biosynthesis”, and “carotenoid biosynthesis” were significantly enriched (Figure 5E).

### Analysis of the Expression Level Changes of Classic Stress-Related Genes in ScDREB5 Transgenic Plants

**FIGURE 6:**
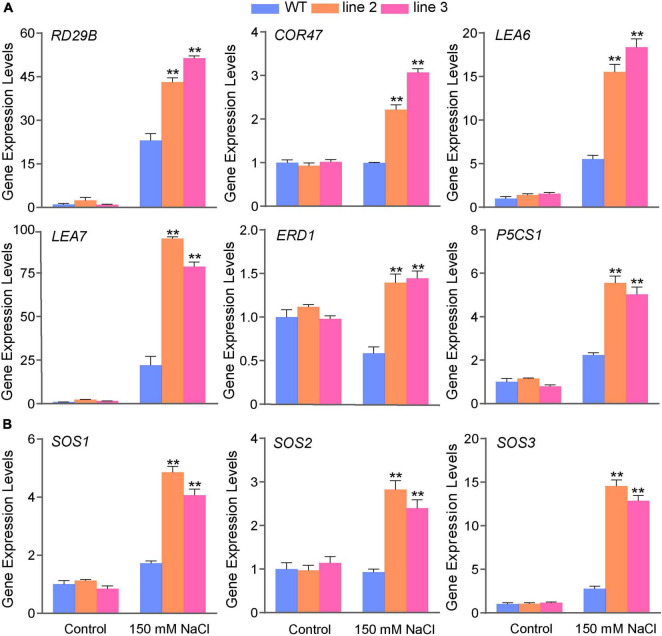
Gene expression level analysis of classic stress-related genes and SOS genes in *ScDREB5* transgenic Arabidopsis response to salt stresses. **(A)** Abiotic stress-responsive genes were analyzed by RT-qPCR in WT and *ScDREB5* transgenic plants under normal and salt stress conditions. **(B)** SOS genes (*SOS1*, *SOS2*, and *SOS3*) were analyzed by RT-qPCR in WT and *ScDREB5* transgenic plants under normal and salt stress conditions. Total RNA was extracted from whole plants. The 2^–ΔΔ*Ct*^ method was used for RT-qPCR normalization. The α*-TUBULIN* (*AT1G50010*) gene of Arabidopsis were used as the reference gene. Values are means ± SE of three replicates. Significant difference compared with WT plants with the same treatment. LSD multiple comparison test was used: *p < 0.05, **p < 0.01.

The transcript abundance of classic stress-related genes, such as *AtRD29B*, *AtLEA*, and *AtSOS*, were significantly upregulated in *ScDREB5* transgenic plants based on RNA-seq (Supplementary Figure 4). RT-qPCR analysis was used to confirm the expression levels of stress-related genes. Under normal conditions, all these genes showed no differences in the expression levels between the transgenic lines and WT plants (Figure 6A); however, after salt stress treatment, the expression of these genes increased significantly. In particular, the *AtLEA7* gene was upregulated approximately 4.3 and 3.6 fold in L2 and L3 compared to the WT, respectively. The SOS pathway plays an essential role in conferring salt tolerance in Arabidopsis (Ji et al., 2013); therefore, we further investigated the expression levels of the SOS pathway genes (*AtSOS1*, *AtSOS2*, and *AtSOS3*). The results showed that the transcripts of *AtSOS1*, *AtSOS2*, and *AtSOS3* were more abundant in response to salinity (both increased more than twofold) (Figure 6B). Overall, these results suggest that the upregulation of classic stress-related genes may partly explain the increased tolerance to salt stress in *ScDREB5* transgenic Arabidopsis.

### Overexpression of ScDREB5 Upregulated Jasmonic Acid Biosynthesis Related Genes Expression and Jasmonic Acid Content Under Salt Stress

**FIGURE 7:**
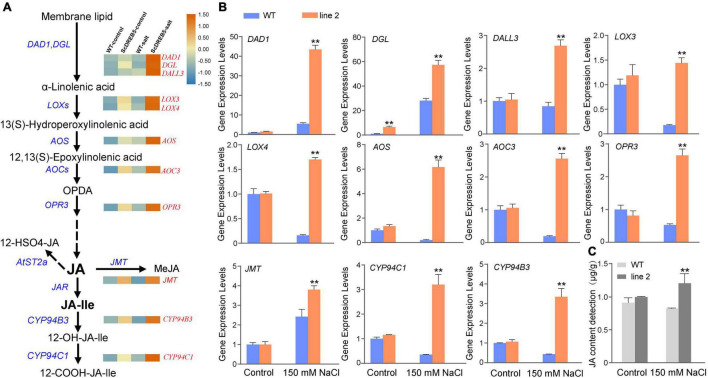
Transcriptome data were used to create heatmaps of the relative expression levels of JA biosynthesis, expression analysis of JA biosynthesis related genes, JA content detection. **(A)** Expression profiling of 11 JA biosynthesis genes which is significantly increased in transgenic plants. These heatmaps were created using TBTools. The FPKM values of the four samples are normalized by a row scale function, the expression levels from low (blue) to high (orange) indicate the minimum and maximum values for the same row. **(B)** Eleven JA biosynthesis related genes were selected for RT-qPCR analysis in WT and *ScDREB5* transgenic plants under normal and salt stress conditions. The α*-TUBULIN* (*AT1G50010*) gene of Arabidopsis was used as the reference gene. **(C)** JA content detection. Values are means ± SE of three replicates. Significant difference compared with WT plants with the same treatment. LSD multiple comparison test was used: *p < 0.05, **p < 0.01.

From the KEGG pathway enrichment analysis, “alpha-linolenic acid metabolism” and “linoleic acid metabolism” had the highest enrichment categories: alpha-linolenic acid and linoleic acid are important precursors of the JA synthesis pathway. To further determine whether *ScDREB5* improves the salt tolerance of transgenic plants by regulating JA, we examined the expression patterns of genes involved in JA biosynthesis based on RNA-Seq data. In total, 19 out of the 24 genes were differentially expressed in transgenic plants (Supplementary Figure 5), and their transcript abundance was higher than that of WT plants under salt stress. The transcript abundance of *PLA1* (*AtDAD1*, *AtDGL*, and *AtDALL3*), *LOX* (*AtLOX3* and *AtLOX4*), *AOC* (*AtAOC3*), *OPR* (*AtOPR1* and *AtOPR3*), and *CYP94* (*AtCYP94C1* and *AtCYP94B3*) were significantly increased in *ScDREB5* transgenic plants (Figure 7A), which were evaluated with RT-qPCR analysis to confirm the abundance change. Under salt stress conditions, all 11 genes had elevated transcript abundances in the transgenic plants compared to the WT plants (Figure 7B); among them, *AtDAD1*, a key gene for JA synthesis, were upregulated eightfold.

We then measured endogenous JA content in WT and transgenic plants under normal and salt stress conditions. Consistent with the gene expression level changes in JA biosynthesis genes, under normal conditions, the JA content of *ScDREB5* transgenic plants was comparable to that of WT plants; however, after salt stress, the JA contents of *ScDREB5* transgenic plants were significantly higher than WT (Figure 7C). These findings, combined with the transcript profiling data, indicated that overexpression of *ScDREB5* enhanced salt stress tolerance in Arabidopsis by regulating JA biosynthesis genes and increasing JA content.

## Discussion

**FIGURE 8:**
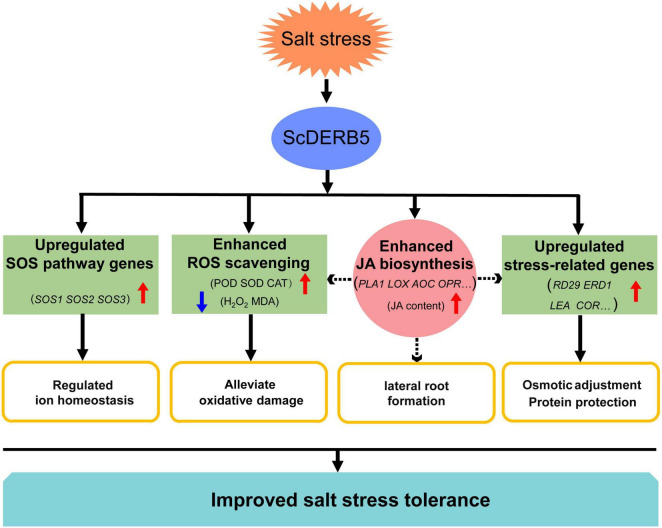
The model of the ScDREB5 regulatory network involved in salt stress responses. Enhanced activities of antioxidant enzymes in the *ScDREB5*-overexpression line resulting in decreased ROS accumulation, improving the expression of SOS pathway and stress-related genes, thus improving salt stress tolerances. *ScDREB5* can activate the expression of JA biosynthesis related genes, modulating the JA content, thus improve salt stress tolerance in Arabidopsis. ↑, ↓ indicate upregulation, downregulation, respectively.

Increasing evidence has shown that A-5 type *DREB* genes in plants play an important role in plant responses to various stresses (Chen et al., 2007, 2009; Liu et al., 2007; Bouaziz et al., 2012; El-Esawi and Alayafi, 2019). In particular, in the desiccation-tolerant moss *S. caninervis*, A-5 *DREBs* were largely expanded and most of the *ScDREBs* showed tolerance to at least one kind of stress in transgenic plants (Li et al., 2016). *ScDREB5*, a novel A-5b type of *DREB* gene from *S. caninervis*, has the highest sequence identity with the *AT1G77640* gene (55%), and the identities of *ScDREB8* and *ScDREB10* were 46 and 54%, respectively. Among these, the function of *AT1G77640* has not yet been reported. *ScDREB8*, belonging to the A-5a type *DREBs*, improved salt stress tolerance in Arabidopsis (Liang et al., 2017). *ScDREB10*, an A-5c *DREB*, enhanced drought and salt tolerances in transgenic Arabidopsis (Li X. et al., 2019). Likewise, an A-5 *DREB, PpDBF1*, from desert moss *Physcomitrella patens* increased salt, drought, and cold tolerances in transgenic tobacco (Liu et al., 2007). These findings indicated that A-5 type DREB proteins in moss species may be the dominant stress response regulators, while A-2 type proteins may be such for Arabidopsis (Li et al., 2017). In this study, *ScDREB5* improved salt resistance in transgenic Arabidopsis (Figure 3). It is well known that salt stress can lead to increased ROS production (Fichman and Mittler, 2020), particularly H_2_O_2_ and O_2_^–^, which can cause oxidative damage with MDA accumulation (Sofo et al., 2015; Yu et al., 2020). It is important for plants to activate antioxidative systems to cope with this oxidative damage (You and Chan, 2015), such as CAT, POD, and SOD, to scavenge ROS. The DAB staining, NBT staining, and MDA and H_2_O_2_ content analysis results showed that *ScDREB5* transgenic lines had lower ROS damage than WT under salt stress (Figures 4A–C), which was consistent with the improved SOD, POD, and CAT activities (Figures 4D–F). The DEG enrichment in the responses to oxygen-containing compounds also indicated that the reactive oxygen system of *ScDREB5* transgenic plants was more active than in that of WT plants under salt stress (Figure 5D). These results suggested that *ScDREB5* alleviated oxidative damage by increasing antioxidant enzyme activities (Figure 8), then decreased H_2_O_2_ levels and MDA content under salt stress. *ScDREB8* and *ScDREB10* also showed enhanced ROS scavenging ability in transgenic Arabidopsis (Liang et al., 2017; Li X. et al., 2019), indicating that it is a common stress tolerance strategy for A-5 type *DREBs*.

Dehydration-responsive element-binding protein transcription factors can induce the expression of a series of downstream genes, such as *RD29*, *LEAs*, *CORs*, *ERDs*, and *P5CS*, which alleviate osmotic damage and protect proline synthesis to enhance abiotic stress tolerances (Agarwal and Jha, 2010; Lata and Prasad, 2011). Similar to other *DREBs*, *ScDREB5* has a highly conserved amino acid composition in the AP2 domain, with 18 out of the 59 identical amino acids in the AP2 domain (Figure 1B). In *DREBs*, amino acids at positions 14 and 19 were primarily valine (V) and glutamic acid (E), and V14 was almost completely conserved in all *DREBs* and played an important role in DNA binding (Sakuma et al., 2002; Li et al., 2017). In this study, transcripts of *AtRD29B*, *AtERD1*, *AtLEA6*, *AtLEA7*, *AtCOR47*/*AtRD17*, and *AtP5CS1* genes were more upregulated in *ScDREB5* overexpression lines than in WT plants under salt stress conditions (Figure 6A), and an increased expression of *AtRD29*, *AtCOR47*/*AtRD17*, and *AtLEA* genes were also found in transgenic *ScDREB8* and *ScDERB10* plants (Liang et al., 2017; Li X. et al., 2019). These results indicated that the genes regulated by an A-5 type *DREB* under stress had relatively high similarity. The SOS signal transduction pathway is very important for ionic homeostasis under salt stress (Yang and Guo, 2018). Wang et al. (2020b) recently reported that the A-2 type *SlDREB2* can directly bind to *SlSOS1* promoters, thus improving the expression level of *SlSOS1* and promoting tomato tolerance to salt stress. *ScDREB8* showed enhanced salt stress tolerance in transgenic Arabidopsis, with no significant difference in the expression levels of the *SOS1*, *SOS2*, and *SOS3* genes (Liang et al., 2017), while their transcript abundances were significantly higher in *ScDREB5* transgenic lines than in WT plants under salt stress (Figure 6B), suggesting that the *ScDREB5* gene may enhance plant salt stress tolerance by regulating the SOS pathway (Figure 8). E19 was conserved in the A-1–A-4 *DREB* groups, while it was variable for the A-5 *DREBs*; *ScDREB5* has a K, whereas *AT1G77640, ScDREB8*, and *ScDREB10* have A, P, and I in this position, respectively (Figure 1B). The change in this amino acid residue within the AP2 domain suggested a different DNA binding preference in A-5 *DREBs*, leading to the expression of different target genes. Our study provides a case where A-5 type *DREBs* may also enhance the expression of SOS pathway genes, thus improving plant salt tolerance.

Dehydration-responsive element-binding protein transcription factors are key regulators of various stress responses, in which they also respond to various plant hormones and improve plant survival under stress conditions (Nolan et al., 2017). For example, ABA and ethylene (ET) are involved in the regulation of abiotic stress in DREB-mediated plants (Xie et al., 2019). JA has been reported to play an important role in plant biotic stress responses, mainly in pathogens and insects (Chini et al., 2016; Bhavanam and Stout, 2021; Bian et al., 2021). Additionally, as cellular hubs for integrating informational cues from the environment, JA is also involved in plant responses to several abiotic stresses, such as salt, drought, heavy metals, and freezing stresses (Wang et al., 2020c). In this study, KEGG pathway analyses of DEG upregulation showed that DEGs were enriched in “alpha-linolenic acid metabolism” and “linoleic acid metabolism” (Figure 5E). The alpha-linolenic acid metabolism pathway can initiate JA biosynthesis (Wasternack and Song, 2017), and linoleic acid can be converted to alpha-linolenic acid through metabolic pathways (Wang et al., 2021), indicating that JA biosynthesis may partially contribute to salt stress tolerance. This hypothesis was supported by the significantly upregulated transcript abundance of JA biosynthesis-related genes in the transgenic lines compared with WT plants under salt stress (Figure 7B), which was also true for the increase in JA content (Figure 7C). JA has been shown to promote the formation of lateral roots (LR; Cai et al., 2014), which are important for water and nutrient acquisition (Yu et al., 2019) and their development plasticity plays an important role in improving plant survival in response to the external environment (Xu et al., 2020). We found that the number of LRs in the transgenic lines with increased JA content was significantly higher than that of WT plants under salt stress (Figure 3F). It may be that *ScDREB5* can activate JA biosynthesis genes, thereby promoting LR formation in transgenic plants exposed to salt stress. Additionally, many transcription factors regulate JA biosynthesis in overexpressed Arabidopsis to improve plant stress tolerance (Verma et al., 2016; Raza et al., 2021). For instance, *VvNAC26* from *Vitis amurensis* has been shown to regulate endogenous JA synthesis, thereby playing a positive role in drought tolerance in transgenic Arabidopsis (Fang et al., 2016), and *IbMYB116* has been shown to enhance drought tolerance by regulating endogenous JA synthesis in transgenic Arabidopsis (Zhou et al., 2019); however, the relationship between *DREBs* and JA has not been extensively studied (Xie et al., 2019). A recent study reported that *ORA47*, an A-5 type *DREB*, plays an important role in JA biosynthesis and signaling (Chen et al., 2016); hence, JA might be involved in the *ScDREB5*-mediated plant salt stress resistance (Figure 8). JA can activate the ROS-scavenging system in plants to improve plant stress tolerance (Lim et al., 2015; Abouelsaad and Renault, 2018; Block et al., 2018). Some reports indicated that the JA contents of transgenic Arabidopsis containing *TaAOC1*, *VaNAC26*, and *IBMYB116* genes increased, accompanied by improved ROS scavenging abilities under stress conditions, but the mechanism is still unclear. *ScDREB5* transgenic lines also showed strong ROS scavenging abilities (Figures 4A–C). After salt stress, H_2_O_2_ levels in *ScDREB5* transgenic Arabidopsis lines (L2 and L3) increased by approximately 1.2 fold and 1.04 fold, respectively, but increased by approximately 2.9 fold in the WT. Therefore, we speculate that the increase in JA content might enhance the ROS scavenging ability of *ScDREB5* transgenic Arabidopsis under salt stress; however, the detailed relationship between ROS scavenging and JA accumulation requires further study. Interestingly, transcript levels of representative salinity-induced genes were induced less with a decrease in endogenous JA content (Mohamed et al., 2015). Li Y. et al. (2019) found that the mediation of the dehydration process by JA occurs *via* the induction of *AtERD1* expression, which is an early response gene in dehydration stress (Nakashima et al., 2010). In our study, the stress marker genes (*AtRD29*, *AtCOR47/AtRD17*, *AtLEA*, *AtERD1*, and *AtP5CS1*) were significantly upregulated in *ScDERB5* transgenic Arabidopsis under salt stress conditions. These results suggest that JA may participate in the *ScDERB5*-mediated process of enhancing salt tolerance by ROS scavenging and modulating stress response genes. In conclusion, JA may act as a crosstalk conjunction for the regulation mechanism in *ScDREB5*-mediated salt stress tolerance.

In conclusion, we identified a novel A-5 type *DREB* gene, *ScDREB5*, from *S. caninervis*, that could improve the salt tolerance of transgenic Arabidopsis by regulating the following processes (Figure 8): **(1) Enhanced ROS scavenging ability:** Overexpression of *ScDREB5* in transgenic plants showed significantly lower ROS damage with significantly lower MDA and H_2_O_2_ contents, and significantly higher SOD, POD, and CAT activities compared to WT under salt stress. **(2) Improved osmotic adjustment and protein protection abilities:** *ScDREB5*-overexpression led to the upregulation of classic stress-related genes (*RD29*, *LEA*, *COR47*, *ERD1*, and *P5CS1*) under salt stress, which can alleviate the osmotic stress caused by salt stress and protein protection. **(3) Regulated ion homeostasis:** Overexpression of *ScDREB5* increased the expression of SOS pathway genes (*SOS1*, *SOS2*, and *SOS3*) in transgenic plants under salt stress, thus regulating ion homeostasis and reducing ion detoxification in plants. **(4) JA is a crosstalk conjunction that may be the core regulation mechanism for ScDREB5-mediated salt stress tolerance:** *ScDREB5* increased endogenous JA content in transgenic Arabidopsis by upregulating JA biosynthesis genes, improving lateral root formation, enhancing ROS scavenging ability, and upregulating stress-related genes under salt stress. However, the detailed biological function of increased JA content and the regulated relationship between *ScDREB5* and SOS pathway genes in *ScDREB5*-mediated salt stress tolerance in transgenic Arabidopsis remains to be elucidated. Future studies should focus on *ScDREB5* mechanisms under salt stress. In summary, we suggest that *ScDREB5* is an excellent candidate gene for improving salt tolerance in plants.

## Data Availability Statement

The datasets presented in this study can be found in online repositories. The names of the repositories and accession number can be found below: http://www.ncbi.nlm.nih.gov/bioproject/798571, PRJNA798571.

## Author Contributions

XL and YW conceived the ideas and designed the experiments. JL, RY, and YL performed the experiments and collected the data. JL reformed all the analyses and wrote the manuscript. All authors contributed to manuscript revision, read, and approved the submitted version.

## Conflict of Interest

The authors declare that the research was conducted in the absence of any commercial or financial relationships that could be construed as a potential conflict of interest.

## Publisher’s Note

All claims expressed in this article are solely those of the authors and do not necessarily represent those of their affiliated organizations, or those of the publisher, the editors and the reviewers. Any product that may be evaluated in this article, or claim that may be made by its manufacturer, is not guaranteed or endorsed by the publisher.

## Footnotes

1
http://www.ncbi.nlm.nih.gov
