# Supplementary material for: The DREB A-5 Transcription Factor ScDREB5 From Syntrichia caninervis Enhanced Salt Tolerance by Regulating Jasmonic Acid Biosynthesis in Transgenic Arabidopsis
Source: Front Plant Sci. 2022 Apr 6;13:857396. doi: 10.3389/fpls.2022.857396 (PMC9019590; doi:10.3389/fpls.2022.857396)
Supplement: Supplementary file 1 [file Data_Sheet_1.docx]

Supplementary Material

# Supplementary Figures


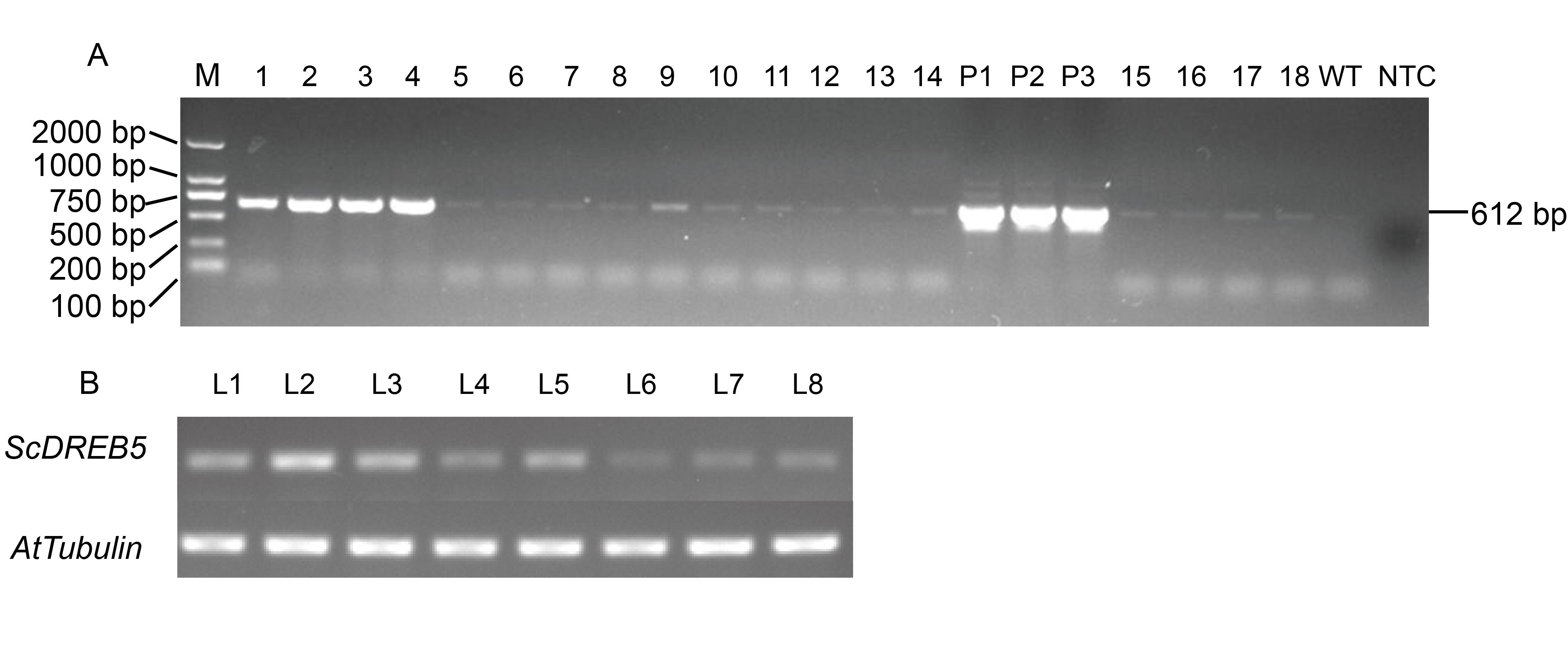


**Supplementary Figure 1 |** The positive tests analysis by PCR **(A)** and expression analysis by RT-PCR **(B)** of *ScDREB5*-overexpression lines. (1-18: positive overexpression-*ScDREB5* lines, P1-P3: positive plasmids; WT: wild type; NTC: no temple control).


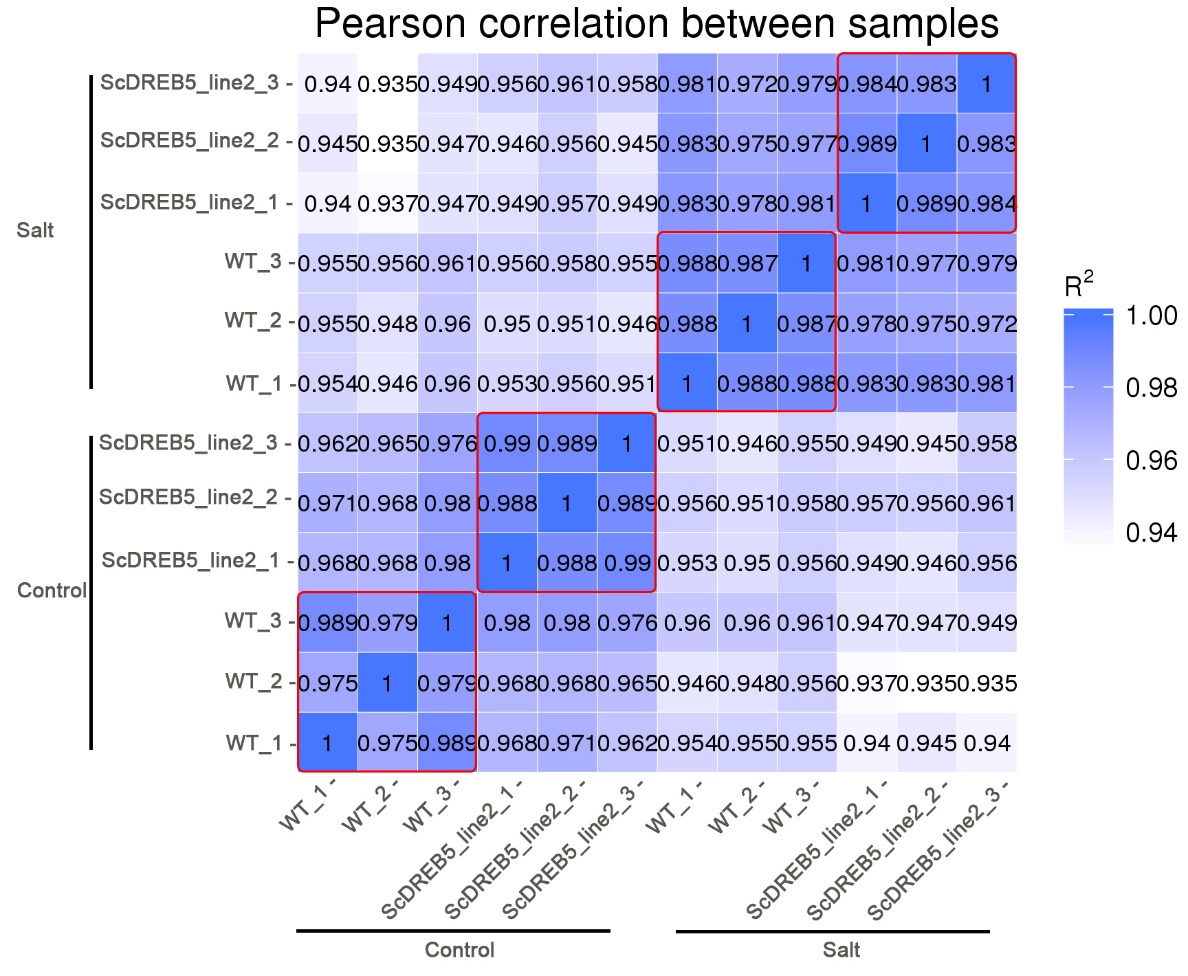


**Supplementary Figure 2 |** The pearson correlation between samples.

**
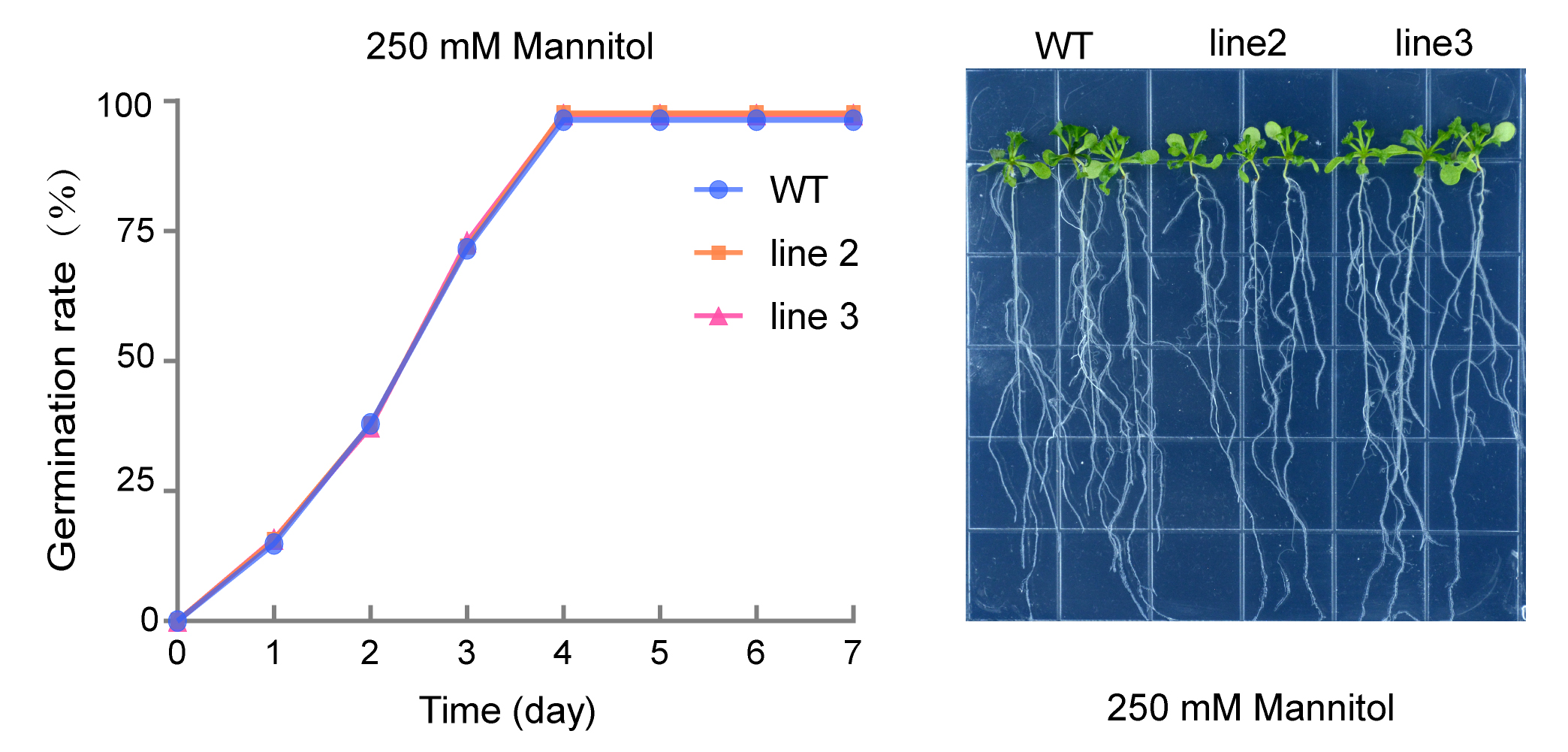
**

**Supplementary Figure 3 |** Germination percentages of WT and *ScDREB5*-transformed seeds under normal and drought stress conditions, calculated as the number of germinated seeds divided by the total number of seeds. Values are means ± SE of three replicates (n = 40-60 seeds).

**
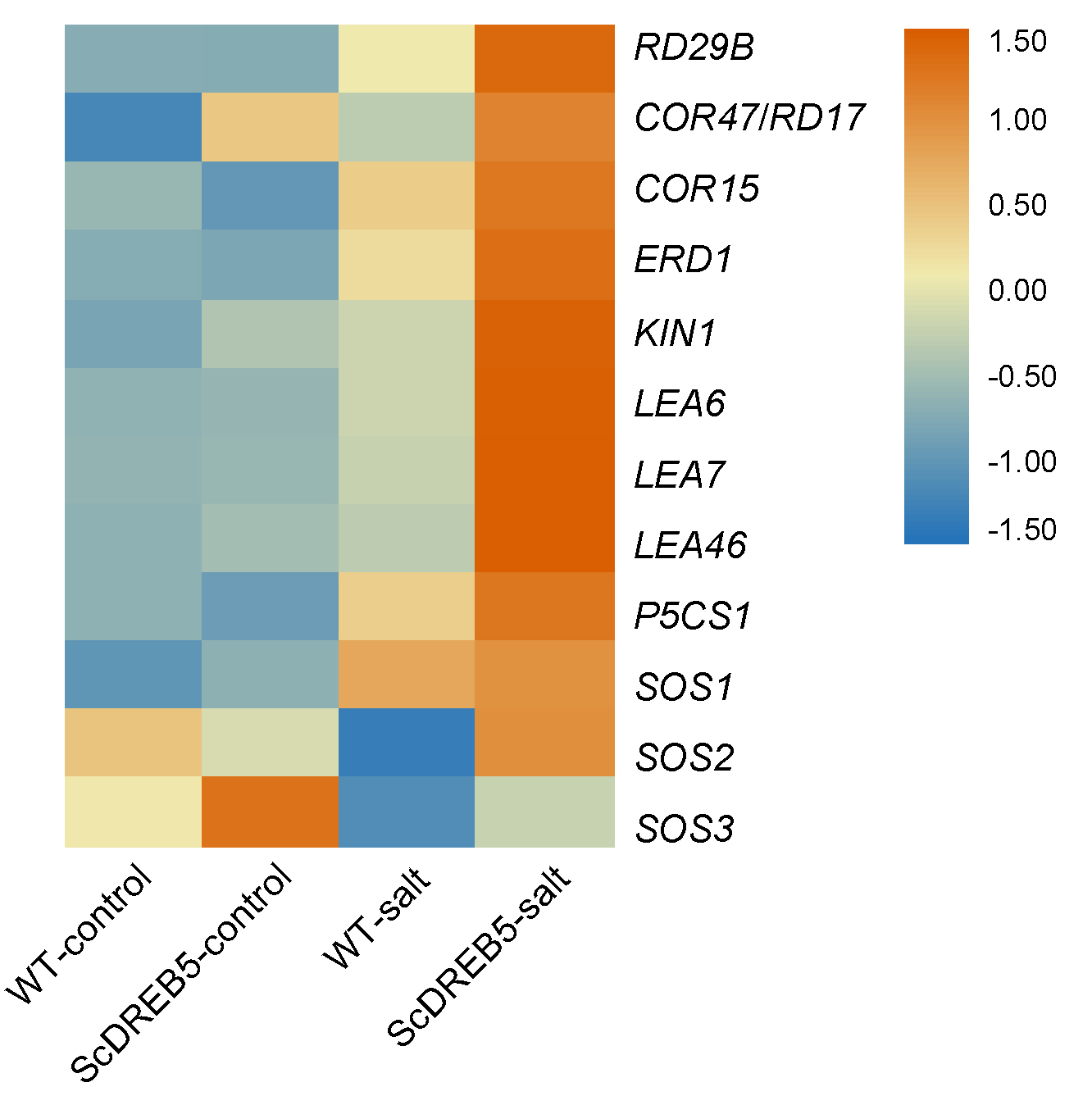
**

**Supplementary Figure 4 |** The heat map analysis of classic stress-related genes under normal and stress conditions. The heat map was created using TBTools. The FPKM values in the four samples are normalized by a row scale function, the expression levels from low (blue) to high (orange) indicate the minimum and maximum values for the same row.

**
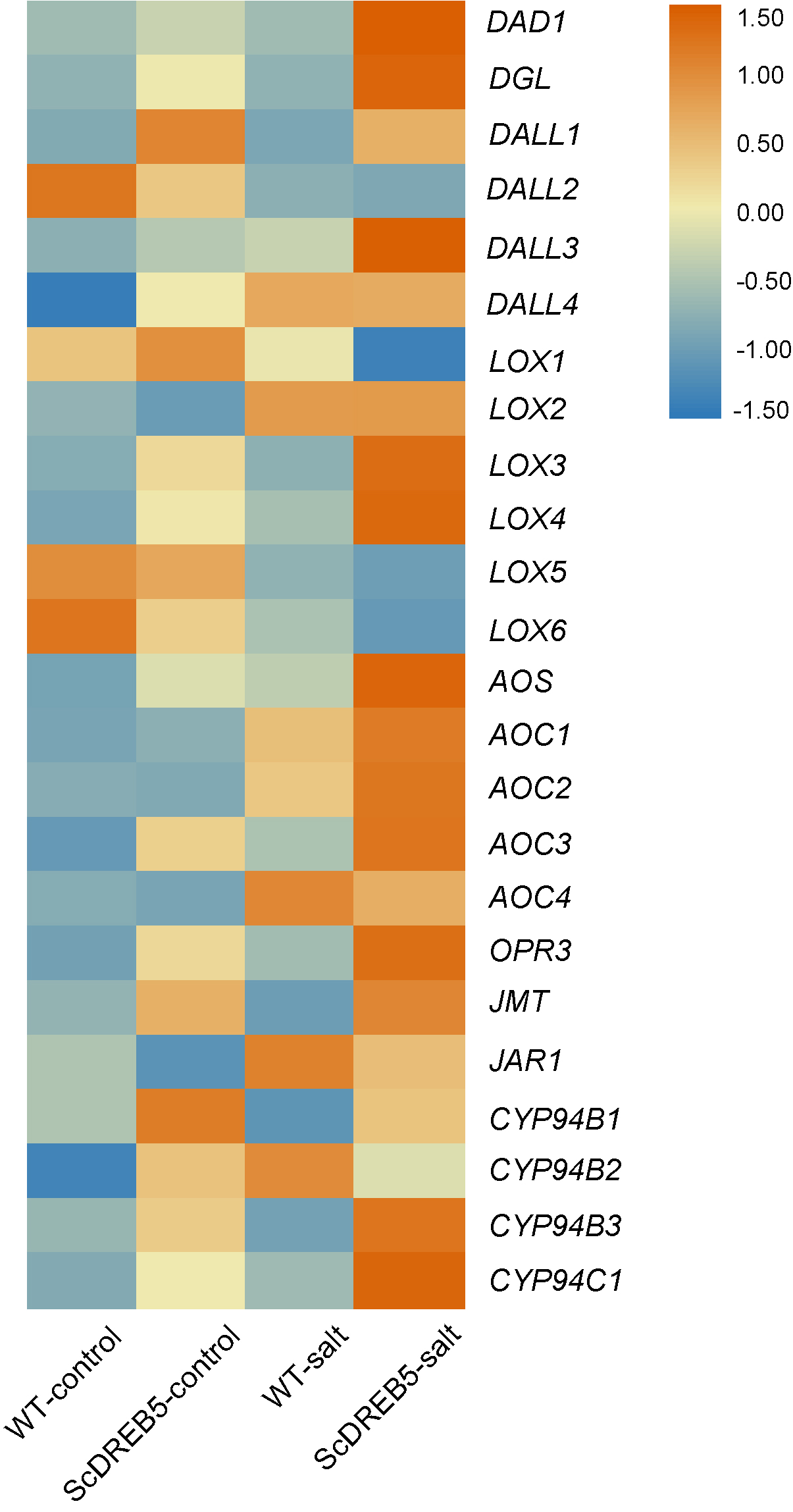
**

**Supplementary Figure 5** **|** The heat map analysis of JA biosynthesis gene expression in WT and transgenic plants under normal and stress conditions. The heat map was created using TBTools. The FPKM values in the four samples are normalized by a row scale function, the expression levels from low (blue) to high (orange) indicate the minimum and maximum values for the same row.

# Supplementary Table

| Assay | Primer name | Sequence (5’ to 3’) |
| --- | --- | --- |
| Subcellular location | pBI121-*ScDREB5*-F | TCTAGACTGGTACCCATGATGGGAGGTGCAGCAGA |
|  | pBI121- *ScDREB5*-R | CTAGTCAGTCGACCCGAAGCTCCACAGCCTGGGT |
| transactivation activity  (Y2H) | pGBKT7-ScSoloist-F | CATGGAGGCCGAATTCATGATGGGAGGTGCAGCAGAGC |
|  | pGBKT7-ScSoloist-R | GCAGGTCGACGGATCCTCAGAAGCTCCACAGCCTGG |

**Supplementary Table 1** **|** List of primers used for vector construction. The vector sequences were underlined.

| Primer name | Sequence (5’ to 3’) | Gene ID |
| --- | --- | --- |
| AtTubulin-F | F: GATGTACCGTGGTGATGTC | AT4G05320 |
| AtTubulin-R | R: GAGCCTCTGAAAATTCTCC |  |
| AtRD29B-F | F: GAAACCAAAGATGAGTCGACAC | AT5G52300 |
| AtRD29B-R | R: TTTTTCGTAAACCGGAGTCAAC |  |
| AtCOR47-F | F: ATGCCAAGACCACTGAAGAGG | AT1G20440 |
| AtCOR47-R | R: AACACAGCGAATGTCCCACTC |  |
| AtLEA6-F | F: GCAGAAGCGAATATGGATATGC | AT1G32560 |
| AtLEA6-R | R: CGGAGGATAAGTCGGATGATAG |  |
| AtLEA7-F | F: TCAAGAGTCCAAAGACAAGACA | AT1G52690 |
| AtLEA7-R | R: GTATATTCAGCTGCATCGTGTG |  |
| AtERD1-F | F: CTTTCTCTATCAGCACGAAACG | AT5G51070 |
| AtERD1-R | R: CGGTGCGATATATTGACAATCC |  |
| AtP5CS1-F | F: AGCTTGATGACGTTATCGATCT | AT2G39800 |
| AtP5CS1-R | R: AGATTCCATCAGCATGACCTAG |  |
| AtSOS1-F | F: ATTTTGATGCAGTCAGTGGATG | AT2G01980 |
| AtSOS1-R | R: GCAAGCAGATTCTAGTCTTTCG |  |
| AtSOS2-F | F: GCGAACTCAATGGGTTTTAAGT | AT5G35410 |
| AtSOS2-R | R: CTTACGTCTACCATGAAAAGCG |  |
| AtSOS3-F | F: CCGGTCCATGAAAAAGTCAAAT | AT5G24270 |
| AtSOS3-R | R: CTCTTTCAATTCTTCTCGCTCG |  |
| AtDAD1-F | F: GTTTACGCTGAAATCGGTAAGG | AT2G44810 |
| AtDAD1-R | R: ACAAACCCGTCTACCAAATGTA |  |
| AtDGL-F | F: CATTTCTTGTGTCCATGACCTC | AT1G05800 |
| AtDGL-R | R: CTTCATTAACCGCCAATTTCGA |  |
| AtDALL3-F | F: CGTTAAAGTCGAATCCGGATTC | AT2G30550 |
| AtDALL3-R | R: GTTCTTCCACTAACCGTTTCAC |  |
| AtLOX3-F | F: TAATCGAAGACTATCCGTACGC | AT1G17420 |
| AtLOX3-R | R: GGTTTGGATAGTAGCGTTCAAC |  |
| AtLOX4-F | F: TAGGAAATCCGGACATATCACG | AT1G72520 |
| AtLOX4-R | R: CTTGGCACATACATCGGTAATG |  |
| AtAOS-F | F: CCTTAAAGGGAAAGCGGATTTC | AT5G42650 |
| AtAOS-R | R: GGCGGATTCTAAGAAAAACTCG |  |
| AtAOC3-F | F: CTCGAGCTTTCTTCTGGAATTG | AT3G25780 |
| AtAOC3-R | R: CGTTGAGCTCATACACGTTTAG |  |
| AtOPR3-F | F: AAGGTGATGCTGATTTGGTTTC | AT2G06050 |
| AtOPR3-R | R: GATAATCCGTGTAGCCAACAAC |  |
| AtJMT-F | F: CATAGTTGACACGATCCACAAC | AT1G19640 |
| AtJMT-R | R: TAAAACTCTGGCAAAGAAGCAC |  |
| AtCYP94C1-F | F: AGATGGATTATTTGCATGCGTC | AT2G27690 |
| AtCYP94C1-R | R: CGTGACTCTAGTCCCACTATTC |  |
| AtCYP94B3-F | F: CATATTCAATGTCGACGGTCAC | AT3G48520 |
| AtCYP94B3-R | R: AAAAGCAAAACTCCTAAGCGAG |  |

**Supplementary Table 2** **|** List of primers for RT-qPCR assay in Arabidopsis.
